# Aminoacyl-tRNA synthetases in medicine and disease

**DOI:** 10.1002/emmm.201100626

**Published:** 2013-02-21

**Authors:** Peng Yao, Paul L Fox

**Affiliations:** Department of Cellular and Molecular Medicine, Lerner Research Institute, Cleveland ClinicCleveland, OH, USA

**Keywords:** aminoacyl-tRNA synthetases (ARSs), human disease, therapeutics, tRNA

## Abstract

Aminoacyl-tRNA synthetases (ARSs) are essential and ubiquitous ‘house-keeping’ enzymes responsible for charging amino acids to their cognate tRNAs and providing the substrates for global protein synthesis. Recent studies have revealed a role of multiple ARSs in pathology, and their potential use as pharmacological targets and therapeutic reagents. The ongoing discovery of genetic mutations in human ARSs is increasing exponentially and can be considered an important determinant of disease etiology. Several chemical compounds target bacterial, fungal and human ARSs as antibiotics or disease-targeting medicines. Remarkably, ongoing exploration of noncanonical functions of ARSs has shown important contributions to control of angiogenesis, inflammation, tumourigenesis and other important physiopathological processes. Here, we summarize the roles of ARSs in human diseases and medicine, focusing on the most recent and exciting discoveries.

## Introduction

Aminoacyl-tRNA synthetases (ARSs) comprise an ancient ubiquitous family of enzymes in all cells from three major kingdoms of life. They catalyse the esterification reactions that link amino acids with cognate tRNAs bearing the correct anticodon triplet to ensure the accurate transfer of information directed by the genetic code (Schimmel, 1987). Generally, the aminoacylation reaction is performed by a two-step process in which amino acids are first activated by ATP, forming an intermediate aminoacyl adenylate, and then transferred to the 3′-end of tRNA to form the aminoacyl-tRNA end-product (Ibba & Soll, 2000). All ARSs contain catalytic and anticodon recognition domains to catalyse the aminoacylation reactions specific for their cognate amino acids. To ensure translational fidelity and maintain normal cellular function, several ARSs have developed editing activities to hydrolyse misactivated amino acids or mischarged tRNAs and prevent insertion of incorrect amino acids during protein synthesis (Schimmel, 2008).

The canonical functions of ARSs, including aminoacylation and editing, are highly conserved throughout the three kingdoms. However, during evolution from prokaryotes to vertebrates, including mammals, certain ARSs acquired appended domains with unique structural characteristics that are neither a part of the enzymatic core nor present in bacterial homologues. These newly evolved domains, generally affixed to the amino or carboxy terminus, are not essential for tRNA charging, but instead are responsible for noncanonical activities unrelated to aminoacylation (Guo et al, 2010), including translation control, transcription regulation, signal transduction, cell migration, angiogenesis, inflammation, and tumourigenesis. Emerging evidence suggests that defects in either canonical or noncanonical ARS functions can cause or contribute to human diseases. Potential associations of ARSs with several types of cancer through aberrant expression and interactions have been described (Kim et al, 2011, 2012b). This review will focus on the most recent and illustrative discoveries about genetic mutations of ARSs in pathology, on ARSs regulating disease-related processes, and the potential use of ARSs as pharmacological targets and therapeutic reagents.

In human cells, two distinct sets of ARSs encoded by separate genes, can be distinguished by their cytoplasmic or mitochondrial localization. According to the standard nomenclature for human ARS genes and proteins, the cytoplasmic forms are abbreviated as the single-letter amino acid code followed by ‘ARS’. For mitochondrial ARS genes, a ‘2’ is appended. Human cells contain 17 cytoplasmic ARS polypeptides (including the bifunctional glutamyl-prolyl-tRNA synthetase, EPRS, responsible for aminoacylation of Glu and Pro; note the exception to the ‘ARS’ nomenclature), 18 mitochondrial ARSs, and 2 dual-localized ARSs present in both cytoplasm and mitochondria (GARS and KARS). In human and other mammalian cells, a large tRNA multi-synthetase complex (MSC) organizes 9 cytoplasmic ARSs and 3 non-enzyme factors, namely MSC p43, p38 and p18 (alternatively known as aminoacyl-tRNA synthetase-interacting multifunctional proteins 1, 2 and 3, or AIMP1, AIMP2 and AIMP3, respectively).

## Charcot–Marie–Tooth disease: An inheritable human disease caused by mutations in cytoplasmic ARSs

**Figure 1:**
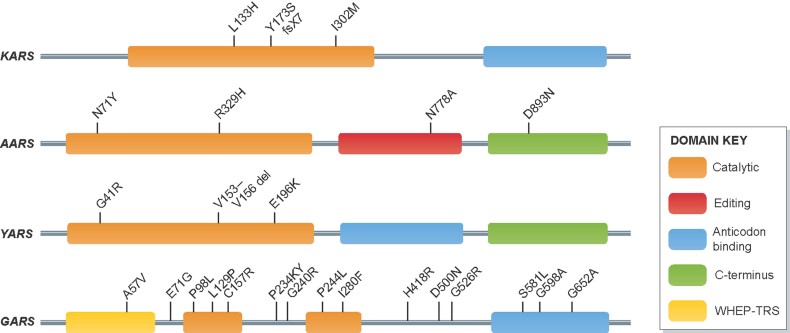
Genetic mutations in human cytosolic ARSs cause CMT disease The domain organization and sites of genetic mutations in human KARS, AARS, YARS and GARS are shown.

Remarkably, to this date all known disease-associated mutations in cytoplasmic ARSs are associated with Charcot–Marie–Tooth (CMT) and related neuropathies (Fig 1). In contrast, mutations in mitochondrial ARSs are associated with a wider variety of syndromes and diseases (Table 1). In 2003, the first human disease-related genetic mutation in an ARS was reported in *GARS* (glycyl-tRNA synthetase) (Antonellis et al, 2003). A mutation in the *GARS* coding region was associated with CMT disease type 2D and distal spinal muscular atrophy type V. CMT disease comprises a genetically and clinically heterogeneous group of autosomal-dominant peripheral neuropathies characterized by progressive degeneration of distal motor and sensory neuron function. Additional dominantly inherited missense mutations in *GARS* have been implicated in CMT disease (Achilli et al, 2009; Banks et al, 2009; Hamaguchi et al, 2010; James et al, 2006; Motley et al, 2010). In this section we focus on mutations in cytoplasmic ARSs associated with CMT disease and their potential underlying mechanisms.

**Table 1:** Compilation of mitochondrial ARS-derived genetic mutations and their connections to human diseases

Gene	Phenotype	Phenotype of corresponding tRNA mutations (Yarham et al, 2010)	Mutations	Refs.	Phenotype OMIM number
*AARS2*	Mitochondrial infantile **CMP**	CPEO, **myopathy**	2: R592W, L155R	Gotz et al (2011)	614096
*DARS2*	LBSL	Myopathy, myoclonic epilepsy and psychomotor progression	15: S45G, R76SfsX5, M134_K165del, C152F, R179H, L239P, R263Q, R263X, E425X, L613F, L626Q, L626V, c.745C>A, c.228-16C>G, c.228-22T>A	Antonellis & Green (2008), Labauge et al (2011), Lin et al (2010), Messmer et al (2011), Yamashita et al (2012)	611105
*EARS2*	**LTBL**	Myopathy, DMDF, encephalomyopathy, respiratory failure, **leukoencephalopathy**, retinitis pigmentosa, exercise intolerance	15: R168G, T426_R427insL, R108W, Y398X, G110S, G204S, E96K, C167Y, G317C, R55H, G224S, M1?, R107H, R516Q, R7X	Steenweg et al (2012)	612799
*FARS2*	Alpers encephalopathy	Myopathy, MELAS, MERRF, exercise intolerance, ataxia, mental deficiency, deafness	2: I329T, D391V	Elo et al (2012)	611592
*HARS2*	Ovarian dysgenesis and **SNHL** of Perrault syndrome	MERRF, MELAS, **deafness**, retinitis pigmentosa	3: L200V, V368L, Del200-211	Pierce et al (2011)	600783
*MARS2*	Spastic ataxia with leukoencephalopathy	Myopathy	3: c.681del268bpfx236X, Dup2/Dup2, Dup1/Dup1	Pierce et al (2011)	609728
*RARS2*	Infantile **encephalopathy**	Myopathy, **encephalomyopathy**	6: L13RfsX3, R291K, I9V, R245Q, Q12R, c.1586 + 3A>T	Cassandrini et al (2013), Edvardson et al (2007)	611523
*SARS2*	HUPRA syndrome	Encephalomyopathy, ataxia, mental deterioration, deafness, myopathy, SNHL, exercise intolerance, MERRF and MELAS	1: D390G	Belostotsky et al (2011)	613845
*YARS2*	**MLASA syndrome**	CPEO, **exercise intolerance**	2: F52L, G46D	Riley et al (2010), Sasarman et al (2012)	613561

Same or related phenotype caused by genetic mutations in corresponding ARS and tRNA pairs are highlighted in bold. CPEO: Chronic progressive external ophthalmoplegia. A type of eye movement disorder, which is characterized by a progressive paralysis of the extraocular muscles. DMDF: Diabetes mellitus and deafness. HUPRA syndrome: Hyperuricemia, pulmonary hypertension, renal failure in infancy, and alkalosis. MERRF: Myoclonic epilepsy and ragged-red fibers. SNHL: Sensorineural hearing loss.

### *YARS* mutations in dominant-intermediate CMT disease

Dominant-intermediate CMT (DI-CMT) is characterized by slow progressive neuropathy, intermediate nerve conduction velocities, axonal degeneration, and demyelination of peripheral motor and sensory neurons. Three dominant mutations (G41R, E196K, V153_V156del) in *YARS* (tyrosyl-tRNA synthetase) are associated with DI-CMT type C (DI-CMTC) (Jordanova et al, 2006). A *Drosophila* model of DI-CMTC was developed in which over-expression of each of the three mutant *YARS* genes, but not the wild-type gene, promotes axon atrophy and impaired motor function, major hallmarks of the human disease (Storkebaum et al, 2009). The loss of tRNA charging activity is not a generally observed feature of DI-CMTC-associated YARS mutant proteins, and is neither necessary nor sufficient to cause the disease phenotype (Froelich & First, 2011). Thus, the DI-CMTC phenotype is not due to haploinsufficiency of the canonical synthetase activity, but more likely is related to a gain-of-function of mutant YARS or possibly to a loss-of-function of an as-yet unknown secondary activity of wild-type YARS.

### KARS and AARS mutations in CMT disease

*KARS* (lysyl-tRNA synthetase) is the third ARS gene associated with CMT disease. Compound heterozygous mutations in the *KARS* gene were identified in a patient with severe neurological symptoms including peripheral neuropathy (McLaughlin et al, 2010). Three variants (p.L133H, p.Y173SfsX7 and p.I302M) were found in *KARS*, with the former two containing loss-of-function mutations that severely inhibit enzymatic activity. An editing-defective, recessive sticky missense mutation (p.A734E) in *AARS* (alanyl-tRNA synthetase) in a mouse model of ataxia was reported in 2006 (Lee et al, 2006). The mutant enzyme mischarges tRNA^Ala^ with Gly or Ser, leads to amino acid misincorporation and protein misfolding, and causes cerebellar Purkinje cell loss and ataxia, but not peripheral axon degeneration. Surprisingly, mutations affecting editing functions have not been observed in patients. Recently, a single family affected by the axonal form of CMT (CMT2) was investigated (Latour et al, 2010). The subjects exhibited sensory-motor distal degeneration secondary to predominant axonal neuropathy and mild demyelination. Sequencing of candidate genes identified a novel mutation in *AARS*, p.R329H. The mutant AARS exhibits reduced aminoacylation activity and might engender reduced translation and consequent neurodegeneration. Moreover, three new pathological mutations were identified in *AARS*: p.N71Y, p.E778A and p.D893N (Lin et al, 2011; McLaughlin et al, 2012; Zhao et al, 2012). The N71Y mutant also exhibited reduced aminoacylation activity; however, the E778A mutant maintained full activity thus providing a second example of a mutated ARS in which a defect in the primary function is unlikely to be responsible for the consequent CMT pathology.

**Glossary**

**Aminoacylation**

The reaction that ARSs catalyse to an attach amino acid to the 3′-end of tRNA and generate aminoacyl-tRNA for protein synthesis.

**Aminoacyl-tRNA synthetase (ARS)**

The family of house-keeping enzymes responsible for aminoacylation of cognate tRNAs.

**Angiogenesis**

The formation of new blood vessels.

**Charcot–Marie–Tooth disease**

A group of inherited motor and sensory peripheral neuropathies.

**CMP**

Heart muscle diseases which result in the deterioration of the function of the myocardium and to heart failure.

**Editing**

The reaction that ARSs catalyse to hydrolyse the misactivated amino acid or mischarged tRNA.

**GAIT**

Gamma-interferon activated inhibitor of translation.

**LBSL**

Leukoencephalopathy with brain stem and spinal cord involvement and lactate elevation.

**MLASA**

Myopathy, lactic acidosis and sideroblastic anemia.

**MSC**

tRNA multi-synthetase complex.

**mtT-DNA**

mitochondrial transfer DNA

**Noncanonical function**

Secondary function unrelated to the primary or canonical function of a protein in a cell, tissue or organ.

**Perrault syndrome**

A rare genetically heterogeneous recessive disorder characterized by ovarian dysgenesis and sensorineural hearing loss or neurological manifestations.

**T_h_17**

T helper 17 cells, a subset of T helper cells that produce interleukin 17 (IL-17) and play a key role in autoimmune diseases.

**UNE-S**

Unique sequence at the N-terminal part of human cytosolic seryl-tRNA synthetase.

### Towards a unifying mechanism of CMT pathology

**Figure 2:**
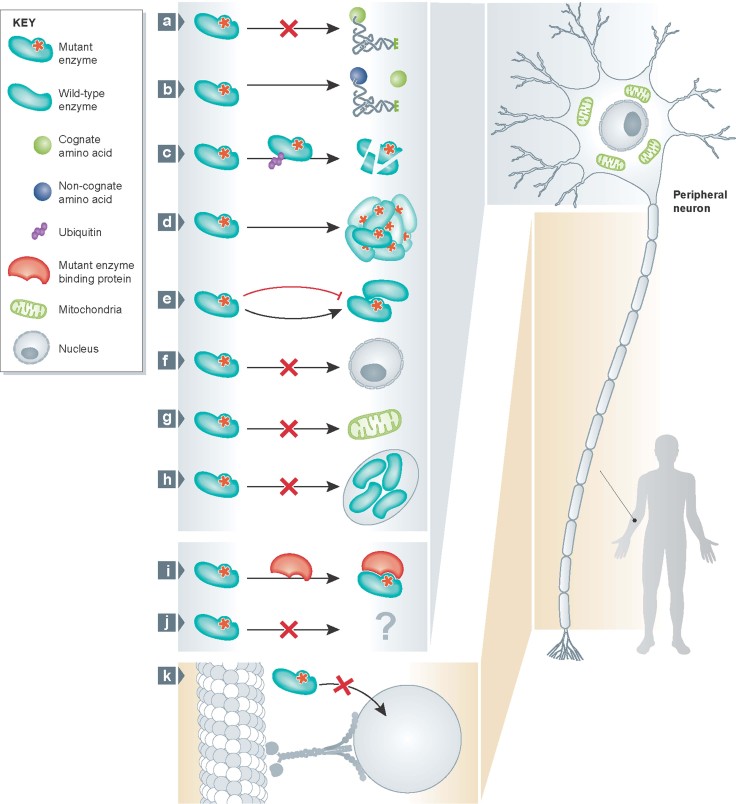
Potential mechanisms underlying CMT caused by ARS mutations Possible etiologic mechanisms of CMT are shown. (a) Defective aminoacylation activity; (b) defective editing activity; (c) destabilization or degradation; (d) aggregate formation; (e) defective dimerization; (f) abnormal nuclear import; (g) abnormal mitochondrial import; (h) abnormal localization in cytosolic granules; (i) gain-of-function by generation of new protein interactions; (j) loss of noncanonical function and (k) defective axonal transport. Genetic mutations in ARSs are indicated (red stars).

An expanding ensemble of genetic mutations in *GARS*, *YARS*, *KARS* and *AARS* associated with CMT and related disorders have been discovered and investigated (Fig 1). However, the molecular and cellular mechanisms linking ARS mutations and the consequent pathology remain unclear. Elucidation of a unifying mechanism underlying CMT caused by mutations at multiple sites within four ARSs remains a major intellectual and experimental challenge. Of the four mutated ARSs, *GARS* is the most extensively studied, leading to several important mechanistic insights. Fifteen mutations in *GARS* have been identified as responsible for clinical phenotypes ranging from CMT neuropathy to a severe infantile form of spinal muscular atrophy. Multiple plausible mechanisms have been proposed (Antonellis & Green, 2008; Motley et al, 2010) (Fig 2), and some have been tested using mouse models (Motley et al, 2011; Stum et al, 2011). Mutations in human cytoplasmic ARSs are primarily located in the catalytic domains responsible for aminoacylation activity. In these cases, a plausible cause is reduced aminoacylation due to mutated synthetic active sites or altered dimerization (Fig 2, mechanism a,e), and consequent defective global protein synthesis. However, multiple findings argue against this mechanism. For example, the Pro to Lys-Tyr mutation (P278KY) in the *Gars*^*Nsf249/+*^ mouse model of CMT2D did not exhibit reduced synthetase activity, and furthermore, heterozygous mice with a single null loss-of-function *GARS* allele exhibited reduced synthetase activity but none of the symptoms of CMT (Seburn et al, 2006). As an example, studies of two dominant mouse models of CMT2D (*Gars*^*Nmf249/+*^ and *Gars*^*C201R/+*^) showed that increased dosage of disease-causing alleles led to more severe neurological phenotypes due to gain-of-function effects that could not be rescued by over-expression of wild-type *GARS*, arguing against a loss-of-function defect (Motley et al, 2011). These observations suggest, at best, a weak relationship between pathological phenotype in CMT and compromised protein synthesis caused by *GARS* mutations. Moreover, several *YARS*, *KARS* and *AARS* mutants associated with CMT retain full catalytic activity, thus providing additional evidence against this mechanism. Reduced aminoacylation activities of some mutant ARSs could intensify the symptoms but might not be sufficient or required. A distinct catalysis-related mechanism could contribute to CMT pathology, namely, mischarging of cognate tRNA and subsequent global incorporation of erroneous amino acids due to compromised editing activity (Fig 2, mechanism b). However, a linkage between defective editing and CMT phenotypes has not been described, in fact, no human disease has been associated with an editing defect. Also, neither misfolding and consequent aggregation nor protein destabilization has been observed for any of the mutant *GARS* associated with CMT pathology (Fig 2, mechanism c,d).

Mutant ARSs might contribute to CMT pathology by at least two alternative mechanisms, namely, altered cellular localization or dysfunctional noncanonical activity. Arguing against the former mechanism, abnormal intracellular distribution of GARS was not detected in mouse central nervous system (CNS) or in peripheral nerves of *GARS* mutant mice (C57BL/6J-*GarsNmf249*/J, *Gars*^*Nmf249/+*^ mice) (Stum et al, 2011). However, given the differences between human and rodent nervous systems, a change in localization of mutant GARS in the CNS or peripheral neurons of human CMT patients, *e.g.*, in nuclei, mitochondria, or cytosolic granules, cannot be formally excluded (Fig 2, mechanism f,g,h). Supporting this possibility is the observation that mast cell KARS undergoes condition-dependent nuclear import and transcriptional regulation of gene expression (Yannay-Cohen et al, 2009), as well as the presence of both GARS and KARS in multiple intracellular compartments including cytoplasm and mitochondria. Furthermore, there is experimental evidence for local protein translation in distal regions of axons supporting the concept that the peripheral motor axon is translationally active (Brittis et al, 2002; Liu-Yesucevitz et al, 2011; Wang et al, 2007). Consistent with this observation, GARS protein is detectable in sciatic axons and Schwann cells in mice. However, the axonic localization is indistinguishable between mutant *Gars*^*Nmf249/+*^ mice and controls (Stum et al, 2011). Notwithstanding these observations in mice, we cannot exclude the possibility that defective axon transport of mutant GARS could compromise local translation and cause axonal degeneration (Fig 2, mechanism k).

GARS secreted from human macrophages exhibits anti-tumourigenic activity (Park et al, 2012). This finding raises the intriguing possibility that GARS may have a noncanonical, axon-specific function unrelated to tRNA charging, and it is a defect in this activity that is a determinant of pathology (Fig 2, mechanism j). Alternatively, a mutation may influence pathology via an injurious gain-of-function unrelated to the canonical activity, for example by acquisition of a new interaction with protein or RNA (Fig 2, mechanism i). A recent structural study of CMT-related GARS mutants suggested that multiple spatially dispersed mutations induced a common conformational change that released inhibition by the α-helical WHEP domain, and exposed a potential gain-of-function interaction surface (He et al, 2011). Despite the above-described mechanisms, each supported by limited evidence, generalized etiologic mechanisms for mutant GARS-directed CMT diseases remain largely unknown.

## Defective mitochondrial ARS aminoacylation activity and disorders of mitochondrial metabolism

Mutations in two mitochondrial ARSs, namely, DARS2 (mitochondrial aspartyl-tRNA synthetase) and RARS2 (mitochondrial arginyl-tRNA synthetase), have been implicated in leukoencephalopathy with brain stem and spinal cord involvement and lactate elevation (LBSL) (Labauge et al, 2011; Lin et al, 2010; Scheper et al, 2007) and infantile encephalopathy (Edvardson et al, 2007), respectively. Similarly, several genetic mutations in EARS2 (mitochondrial glutamyl-tRNA synthetase), MARS2 (mitochondrial methionyl-tRNA synthetase) and FARS2 (mitochondrial phenylalanyl-tRNA synthetase) cause leukoencephalopathy with thalamus and brainstem involvement and high lactate (LTBL) (Steenweg et al, 2012), spastic ataxia with leukoencephalopathy (Bayat et al, 2012) and Alpers encephalopathy (Elo et al, 2012), respectively. An expanding ensemble of genetic mutations in mitochondrial ARSs has been identified in a variety of pathological contexts (Table 1).

### Mutation of *YARS2* causes myopathy, lactic acidosis and sideroblastic anemia (MLASA) syndrome

Mitochondrial respiratory chain disorders are heterogeneous and among the most common inborn defects of metabolism, with an incidence of more than 1 in 8000 births. MLASA is a mitochondrial respiratory chain disorder characterized by progressive exercise intolerance and sideroblastic anemia. A pathogenic, recessive mutation (p.F52L) in the *YARS2* gene (mitochondrial tyrosyl-tRNA synthetase) has been identified (Riley et al, 2010). Importantly, recombinant mutant YARS2 protein exhibited 9-fold lower tyrosylation activity *in vitro* compared to wild-type enzyme. Reduced aminoacylation activity of the mutant enzyme could cause decreased mitochondrial protein synthesis and consequent mitochondrial respiratory chain dysfunction.

### *HARS2* mutations cause ovarian dysgenesis and sensorineural hearing loss in Perrault syndrome

Perrault syndrome is a rare, genetically heterogeneous, recessive disorder characterized by ovarian dysgenesis and sensorineural hearing loss, or other neurological manifestations. In a non-consanguineous family with five affected siblings, compound heterozygosity for mutations in *HARS2* (mitochondrial histidyl-tRNA synthetase) at two highly conserved amino acids (p.L200V and p.V368L), and an alternative splice-derived deletion of 12 in-frame codons, were shown to be causative (Pierce et al, 2011). Point-mutant HARS2 V368L protein exhibited reduced aminoacylation activity, and the deletion mutant lost essentially all activity. RNAi-mediated knockdown of *hars-1* in *Caenorhabditis elegans* caused severe gonadal defects, *e.g.*, the absence of oocytes or fertilized eggs, resulting in compromised fertility. Possibly, Perrault syndrome is related to aberrant mitochondrial translation and gonadal dysgenesis.

### *SARS2* mutations cause hyperuricemia, pulmonary hypertension, renal failure in infancy and alkalosis (HUPRA) syndrome

A new multisystemic mitochondrial cytopathy was diagnosed in three infants from consanguineous Palestinian kindred. The key clinical findings were tubulopathy, pulmonary hypertension and progressive renal failure in infancy. The patients experienced severe feeding difficulties and developmental delay and died of multi-organ failure, respiratory insufficiency, or refractory pulmonary hypertension. The enzymatic activities of the respiratory chain complexes I–IV were reduced in mitochondria from patients' muscle specimens. A pathogenic, recessive mutation (p.D390G) in the enzymatic active site of SARS2 (mitochondrial seryl-tRNA synthetase) was identified (Belostotsky et al, 2011). In peripheral lymphocytes obtained from patients, the D390G mutation exhibited lower serylation activity for the isoacceptor tRNA^Ser^_AGY_ but not for tRNA^Ser^_UCN_, and the uncharged tRNA is destabilized and degraded. This mutation is the first example of a defective ARS differentially recognizing two tRNA isoacceptors leading to translational inefficiency.

### *AARS2* mutations in mitochondrial infantile cardiomyopathies

Infantile mitochondrial cardiomyopathies (CMPs) are fatal disorders occurring during the neonatal period or first year after birth. The exome of a hypertrophic mitochondrial CMP infant with cardiac respiratory chain deficiency, who died at 10 months of age, was sequenced. A homozygous missense mutation (p.R592W) was identified in *AARS2* (mitochondrial alanyl-tRNA synthetase) (Gotz et al, 2011). Two siblings from an unrelated family, both of whom died perinatally of hypertrophic CMP, harboured the same mutation, compound heterozygous with a second missense mutation (p.L155R). The mutations in *AARS2* cause perinatal or infantile CMP with near-total deficiency of mitochondrial respiratory chain complexes I and IV in the heart. Computational modelling of AARS2 structure suggested that these mutations might affect aminoacylation or editing functions.

To date, all mitochondrial ARSs bearing genetic mutations causing human diseases exhibit compromised aminoacylation activity, and effects on noncanonical functions unrelated to translation have not been demonstrated. Thus, the etiology underlying human mitochondrial disorders might be restricted to defective enzymatic activities. These observations raise an important question: Given that genetic mutations from mitochondrial ARSs affect fundamental mitochondrial functions, for example, translational repression of mitochondrial proteins and consequent respiratory chain defects, why do they cause a diversity of disease phenotypes in distinct affected tissues? Possibly, the threshold amount of an ARS required for optimal enzymatic activity differs for each individual ARS and tissue combination. Thus, a change in the activity of a mutant ARS might be tolerated in some tissues but not in others. Alternatively, there might exist tissue-specific proteins that bind the mutant ARS, leading to disease phenotypes by inhibiting the enzymatic activity, or by diminishing potential noncanonical functions.

Human mitochondrial DNA encodes 22 mitochondrial tRNAs (Yarham et al, 2010). Inheritable mutations in mitochondrial tDNA (mtT-DNA) are hot spots of pathology causing a wide spectrum of clinical phenotypes including neuromuscular disorders. Intriguingly, genetic mutations in either member of at least five cognate ARS-tRNA pairs (AARS2-mtT-DNA^Ala^, EARS2-mtT-DNA^Glu^, HARS2-mtT-DNA^His^, RARS2-mtT-DNA^Arg^ and YARS2-mtT-DNA^Tyr^) cause the same or similar pathological phenotypes, possibly due to a common loss-of-activity mechanism (Table 1). However, the pathological outcomes of mutations in other pairs do not appear to overlap (*i.e.* DARS2-mtT-DNA^Asp^, FARS2-mtT-DNA^Phe^, MARS2-mtT-DNA^Met^ and SARS2-mtT-DNA^Ser^). Mutations at distinct sites in the same mtT-DNA gene sometimes lead to multiple disease types, possibly due to diverse effects on tRNA synthesis, maturation, accepting activity, or stability. In contrast, mutations in mitochondrial ARSs impact aminoacylation activity without affecting production, processing or turnover of cognate tRNAs, possibly accounting for the phenotype mismatch between mutations of ARS-tRNA pairs.

## ARSs as pharmacological targets against pathogenic microorganisms and in autoimmune disease

### Mupirocin targets aminoacylation site of bacterial IARS

**Figure 3:**
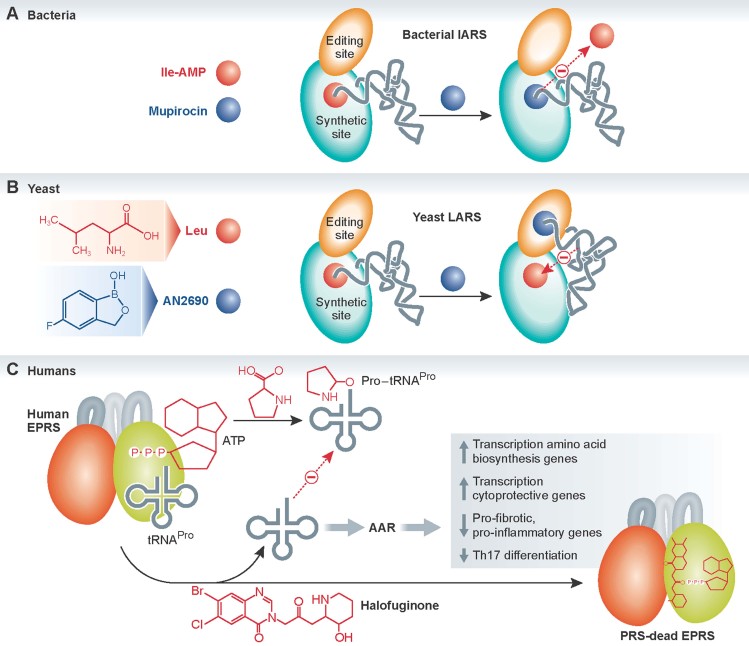
ARSs as drug targets The anti-bacterial reagent mupirocin targets the bacterial IARS synthetic active site by blocking Ile-AMP binding.The anti-fungal reagent AN2690 targets the yeast LARS editing active site by boron-mediated trapping of tRNA^Leu^.The anti-fibrotic reagent halofuginone targets synthetic active site of PRS in human EPRS, triggering the AAR pathway and inhibiting T_h_17-cell differentiation.

Antibiotic resistance of pathogenic bacteria is a major threat to public health and provides a rationale for screening natural products to seek new antibiotic therapies. Clinical effectiveness and low side-effects of ARS inhibitors demand extremely high selectivity for bacterial *versus* human ARS targets. ARSs exhibit multiple characteristics potentially advantageous for targets, including (i) high evolutionary divergence of prokaryotic and eukaryotic ARSs; (ii) the full complement of bacterial ARSs provides 20 distinct targets; (iii) a wealth of structural information on both bacterial and eukaryotic ARSs and (iv) an abundance of known natural inhibitors that can be used for starting points for drug development. Mupirocin (pseudomonic acid) is currently the only ARS inhibitor commercially used as an antibiotic. It is a natural product isolated from *Pseudomonas fluorescens* that inhibits eubacterial and archaeal IARS catalytic activity, and is topically effective against gram-positive pathogens (Silvian et al, 1999). The antibiotic inhibits the enzymatic activity of IARS by blocking the synthetic binding site of the intermediate Ile-AMP (Fig 3A) (Nakama et al, 2001). Most antibiotics under development to target ARS active sites are natural or synthetic compounds mimicking the aminoacyl-AMP moieties to repress aminoacylation activity of the corresponding ARSs.

### AN2690 targets editing site of fungal LARS

AN2690 (5-fluoro-1,3-dihydro-1-hydroxy-2,1-benzoxaborole) is a broad-spectrum, anti-fungal therapeutic agent specifically developed for topical treatment of onychomycosis (Baker et al, 2006). Onychomycosis, commonly referred to as ‘nail fungus’, is primarily caused by dermatophytes, fungi that infect the skin or nails. AN2690 inhibits the essential fungal enzyme, LARS, and was designed with three distinguishing characteristics, namely, enhanced nail penetration, absence of systemic side effects, and potent mechanism-based antifungal activity (Rock et al, 2007). The inhibition of protein synthesis by AN2690 leads to specific repression of fungal cell growth (Yao et al, 2008), thereby eliminating the infection. Mechanistically, AN2690 inhibits synthesis of leucyl-tRNA^Leu^ by formation of a stable boron-mediated tRNA^Leu^-AN2690 adduct in the editing site of LARS and consequently blocking protein synthesis (Fig 3B). Importantly, these findings have established the editing site as a bona fide target for ARS inhibitors. AN2690 did not produce systematic side-effects during phase I and phase II clinical trials (http://www.anacor.com). The high specificity of AN2690 for the fungal enzyme is possibly due to inefficient penetration of human cell plasma membranes or inaccessibility of LARS in the MSC.

### Halofuginone targets human EPRS, activates amino acid response (AAR) pathway and inhibits T_h_17-cell differentiation

Febrifugine is a bioactive natural product extracted from roots of the hydrangea *Dichroa febrifuga Lour* and used in traditional Chinese medicine. Febrifugine derivatives have been used to treat malaria, cancer, fibrosis and inflammatory disease. T helper 17 (T_h_17) cells, a T helper cell sub-class characterized by robust interleukin 17 (IL-17) production, have a key role in autoimmune diseases (Maddur et al, 2012). Halofuginone (HF), a low-toxicity, halogenated febrifugine derivative, activates the amino acid response (AAR) pathway and inhibits the development and progression of T_h_17-driven autoimmunity in a mouse model of multiple sclerosis (Fig 3C) (Keller et al, 2012). HF is in clinical trials as a therapeutic against cancer and fibrotic disease. The HF target in human cells is human EPRS. HF acts as an ATP-dependent, prolyl adenylate-mimetic that specifically inhibits PRS aminoacylation activity. At an appropriate dose that does not cause global translational arrest, HF triggers the AAR pathway, inhibits T_h_17 differentiation, and induces antifibrotic activities in fibroblasts. The AAR pathway is triggered by amino acid starvation, and results in an increase of transcription factor ATF4, which in turn limits or increases the production of other downstream target proteins. The novel pharmacological mechanism suggests that the AAR pathway might be an important target for drugs accelerating the resolution of inflammation. HF has long been applied as an anti-parasitic agent, and the recent realization of its activity as an anti-autoimmune response inhibitor expands its potential clinical utility. It remains an open question whether other ARS-targeting antibiotics with low efficiency for human enzymes can be developed into therapeutic agents targeting human disease.

## Noncanonical functions of ARSs in regulating gene expression and cellular functions related to disease

**Figure 4:**
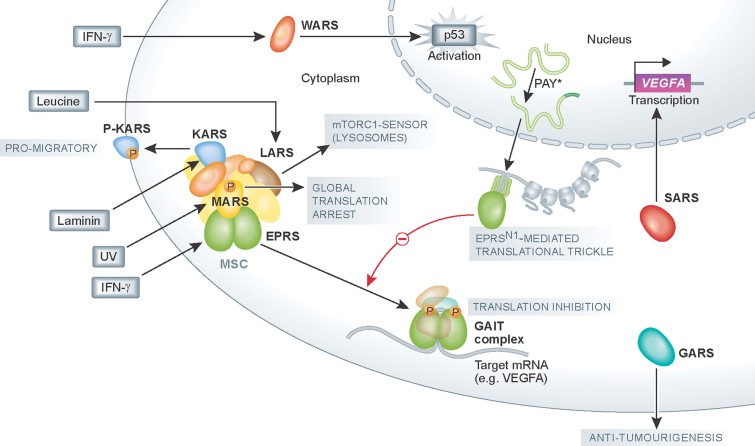
Noncanonical functions of ARSs in regulating cell functions Recent discoveries of noncanonical functions of human ARSs and their underlying mechanisms. IFN-γ-induced release of EPRS from the MSC and assembly of the GAIT complex that mediates translational silencing of inflammation-related mRNAs. UV-triggered phosphorylation of MARS inhibits global translation and activates tumour suppressor MSC p18. SARS directs transcriptional repression of *VEGFA* during vertebrate vascular development. WARS mediates crosstalk between IFN-γ and p53 signalling pathway to activate p53. LARS acts as a leucine sensor in mTORC1 signalling to regulate cell size and autophagy. Laminin-stimulated membrane localization of KARS promotes cell migration. Human macrophages secret GARS to suppress tumour growth. See details in the text.

The noncanonical functions of ARSs beyond global translation have been reviewed elsewhere (Guo et al, 2010; Park et al, 2008). In mammalian cells, three non-enzyme factors that reside with nine of the ARSs in the MSC, *i.e.* MSC p43, p38 and p18, also exhibit cellular activities beyond translation outside of the MSC. The physiological and pathological implications of these non-synthetase components of the MSC have been reviewed elsewhere (Park et al, 2005b, 2010). Here, we focus on recent advances in our understanding of noncanonical functions of human cytoplasmic ARSs (Fig 4).

### Phosphorylated EPRS directs gene-specific translational silencing of inflammation-related mRNAs

Macrophages function in innate immunity in vertebrates by phagocytosing pathogens and cellular debris, and by releasing cytotoxic proteins as a defence against infection. Over-accumulation of these toxic agents can be detrimental to host tissues and organisms, and contribute to chronic inflammatory diseases. The EPRS-bearing GAIT (gamma-interferon-activated inhibitor of translation) complex suppresses translation of a ‘regulon’ of inflammation-related mRNAs, including ceruloplasmin (*Cp*), vascular endothelial growth factor-A (*VEGF-A*), and several chemokine receptors and ligands among others (Mukhopadhyay et al, 2008; Ray et al, 2009; Vyas et al, 2009). The complex might participate in the ‘resolution of inflammation’ by restricting production of injurious proteins (Sampath et al, 2004). EPRS is the unique GAIT constituent that binds the GAIT RNA element in the 3′-UTR of target mRNAs. In human myeloid cells, interferon-γ induces sequential phosphorylation of Ser^886^ and Ser^999^ of EPRS in the noncatalytic linker connecting the synthetase cores (Arif et al, 2009). Ser^886^ phosphorylation is required for binding NSAP1 (NS1-associated protein 1) to form an inactive pre-GAIT complex. Ser^999^ phosphorylation directs the formation of the active GAIT complex that binds initiation factor eIF4G and blocks recruitment of the pre-initiation complex. Unexpectedly, a C-terminus truncated form of EPRS, termed EPRS^N1^, was found to bind GAIT target mRNAs and prevent binding of the functional GAIT complex, thereby acting as a dominant-negative inhibitor of GAIT activity. This imposes a ‘translational trickle’ of target protein expression at basal levels required for tissue well-being (Yao et al, 2012). Genetic or condition-dependent defects in EPRS have not been reported; however, pathological dysregulation of another GAIT constituent protein, ribosomal protein L13a, by oxidized lipoproteins disrupts the GAIT system, potentially contributing to progression of chronic inflammatory disorders (Jia et al, 2012).

### MARS regulates global translation and tumour suppressor activity of MSC p18

Human MARS has an especially critical role in translation initiation by transferring Met to initiator tRNA_i_^Met^. MARS is a component of the MSC and provides a docking site for MSC p18 in the cytoplasm (Kwon et al, 2011). MSC p18 is a potent tumour suppressor that translocates to the nucleus for DNA repair upon DNA damage (Park et al, 2005a), and also regulates translational initiation by mediating the delivery of charged tRNA_i_^Met^ to the initiation complex (Kang et al, 2012). UV irradiation induces phosphorylation of MARS at Ser^662^ by GCN2 (general control nonrepressed-2), releasing tumour suppressor MSC p18 for nuclear re-localization. Phosphorylated MARS exhibits significantly reduced catalytic activity due to diminished tRNA_i_^Met^ binding, and a consequent repression of global translation. Interestingly, UV-stress-induced MARS phosphorylation, and subsequent global translational repression, mirrors IFN-γ-induced EPRS phosphorylation and transcript-selective translational silencing program, revealing a noncanonical theme common to these and possibly other synthetases.

### SARS mediates transcriptional repression during vascular development

In a genetic screen in zebrafish, mutations in the *sars* gene were found that affect vascular development and maintenance, including aortic arch vessel dilatation, aberrant hindbrain capillary patterning, abnormal intersomitic vessels, or disorganized vessels with abnormal branching of established intersegmental vessels (Fukui et al, 2009; Herzog et al, 2009). Phenotypic rescue by over-expression of an enzymatically inactive form of *sars* T429A and knockdown of *vegf receptor* or its ligand *vegf-a*, suggest that a noncanonical function of SARS in repression of VEGF-A expression contributes to vascular development. Injections of highly homologous human *SARS* mRNA rescued the vascular branching phenotype in *sars* mutant-bearing zebrafish suggesting that the regulatory function of SARS in vascular development might be conserved between zebrafish and human. The UNE-S (Unique-S) domain, appended to SARS at the C-termini and containing nuclear localization signal (NLS), was identified as an essential domain for shuttling the enzyme into nucleus and repressing *VEGF-A* mRNA transcription, thereby permitting normal vascular development (Xu et al, 2012). It remains an open question whether SARS directly interacts with *VEGF-A* gene promoter and modulates its transcription, or acts by an indirect mechanism.

### WARS mediates crosstalk between IFN-γ and p53 signalling pathways

WARS exhibits a noncanonical regulatory function for activation of p53 (Sajish et al, 2012). IFN-γ upregulates WARS and facilitates formation of a nuclear trimeric complex with the catalytic subunit of DNA-dependent protein kinase (DNA-PKcs) and poly(ADP-ribose) polymerase 1 (PARP-1). WARS bridges the N-terminal domain of PARP-1 with the kinase domain of DNA-PKcs. Within the complex, PARP-1 catalyses poly(ADP-ribosyl)ation of DNA-PKcs, thereby activating the kinase which in turn activates the tumour suppressor p53 by phosphorylation at Ser^15^. Over-expression of WARS by itself activates p53 indicating that other IFN-γ signalling pathways are not required. These results suggest that the antiproliferative and antiangiogenic responses induced by IFN-γ-mediated activation of p53 are triggered in part by upregulation and nuclear localization of WARS.

### LARS is a leucine sensor in mTORC1 signalling

The protein kinase mTOR (mammalian target of rapamycin) controls various critical cellular processes including cell growth, autophagy, protein synthesis and metabolism. Activation of the mTOR signalling pathway contributes to multiple human pathologies including cancer and tissue hypertrophy. Pharmacological inhibitors of mTOR are used clinically to prevent graft rejection and restenosis after angioplasty. mTOR complex 1 (mTORC1) is activated by metabolic inputs such as elevated Leu and other amino acids (Wang & Proud, 2011). Recently, human LARS was identified as an important intracellular sensor for mTORC1 signalling through its Leu activation activity, independent of tRNA^Leu^ (Han et al, 2012). In the presence of Leu, LARS translocates to the lysosome, interacts with mTORC1, Raptor (regulatory associated protein of mTOR) and the GTP-bound form of RagD, and promotes GTP hydrolysis and mTORC1 activation. Knockdown of LARS reduces cell size and increases autophagy. The interaction between RagD and LARS is mediated by the non-catalytic C-termini of both proteins. Consistent with these findings, yeast LARS was identified as a sensor for TORC1 activation (Bonfils et al, 2012), suggesting evolutionary conservation of this important noncanonical function. It remains to be determined whether elevated LARS in cancer cells causes hyperactivation of mTORC1 signalling with pathological consequences such as tumourigenesis (Shin et al, 2008).

### Membrane complex of KARS and laminin receptor promotes cell migration

Human KARS exhibits regulatable cellular localization and multiple functions beyond translation including transcription control upon nuclear import (Yannay-Cohen et al, 2009) and cytokine signalling after release into the extracellular space (Park et al, 2005c). Recently, a new membrane translocation event for KARS was reported (Kim et al, 2012a). Upon laminin stimulation, KARS is phosphorylated at Thr^52^ residue by p38 MAP kinase, dissociates from the MSC, and translocates to the plasma membrane where it assembles into a complex with the 67 kDa laminin receptor (67LR). KARS inhibits ubiquitin-dependent degradation of 67LR, thereby promoting laminin-induced cell migration. This pro-migratory function of KARS suggests a possible role in tumour cell metastasis.

### GARS as an endogenous anti-tumourigenesis reagent

Human GARS is released from macrophages in response to tumour-derived Fas ligand (Park et al, 2012). GARS binds ERK-activated tumour cells through cadherin (CDH)6 and induces release of phosphatase 2A (PP2A) from CDH6. Activated PP2A inhibits ERK signalling through ERK dephosphorylation, and induces apoptosis. *In vivo* administration of GARS strongly suppresses tumour growth in a mouse model, accompanied by CDH6 induction and ERK activation. Thus, GARS has potential applicability in anti-tumour therapy.

## Concluding remarks

ARSs have a triple-faceted relationship with human health and disease. First, the loss-of-function of an ARS resulting from genetic mutations can cause a broad spectrum of diseases. Human LARS can correct mitochondrial dysfunctions caused by tRNA^Leu^_UUR_ A3243G mutation-related neurodegenerative disorder, MELAS syndrome (Li & Guan, 2010). This finding provides a step toward therapeutic interventions for human disorders by introducing exogenous ARSs. Second, antibiotic-directed inhibition of ARS activities from lower species, including bacteria and fungi, can treat infectious diseases. Interestingly, targeting specific functions of human ARSs can have beneficial clinical output rather than cytotoxic effect, for example, for treatment of autoimmune diseases. Third, elucidation of emerging noncanonical functions of human ARSs will provide unique opportunities for therapeutic intervention. Because many therapeutic reagents with high potency also exhibit adverse side effects, *e.g.*, elevated risk of coronary heart disease by nonsteroidal anti-inflammatory drugs, and disturbance of blood vessel maintenance by anti-VEGF-A inhibitors, the application or stimulation of natural secretory or endogenous ARSs (*e.g.*, GARS and EPRS), might provide a new set of physiologic extracellular or intracellular pathways as basis for developing novel therapeutics with minimal side-effects.

**Pending issues**

Clarification of molecular mechanisms underlying ARS mutation-derived CMT disease.

Development of ARS-based therapeutic reagents to treat CMT and mitochondrial ARS-defective diseases.

Systematic exploration of the physiopathological relevance of noncanonical function of human ARSs.

Development of therapeutic strategies based on secretory ARSs, endogenous ARSs, or ARS antibodies.
